# Optical coherence tomography angiography in glaucoma: a mini-review

**DOI:** 10.12688/f1000research.11691.1

**Published:** 2017-09-14

**Authors:** Kelvin H. Wan, Christopher K. Leung

**Affiliations:** 1 Department of Ophthalmology, Tuen Mun Eye Center and Tuen Mun Hospital, Hong Kong, China; 2 Department of Ophthalmology and Visual Sciences, The Chinese University of Hong Kong, Hong Kong, China

**Keywords:** optical coherence tomography angiography, glaucoma, retina, optic nerve head, vascular abnormalities, optical imaging

## Abstract

The advent of optical coherence tomography angiography (OCT-A) provides a new opportunity to visualize the retinal vasculature in a non-invasive and dye-free manner which may help identify vascular abnormalities in glaucoma. While a reduction in retinal and optic nerve head vessel densities and blood flow indexes measured by OCT-A has been demonstrated in patients with glaucoma in many studies, it is unclear whether OCT-A provides additional information for the detection and monitoring of glaucoma compared with OCT measurements such as retinal nerve fiber layer thickness, neuroretinal rim width, and ganglion cell inner plexiform layer thickness. Longitudinal studies are needed to elucidate whether vascular abnormalities detected by OCT-A are a cause or a consequence of optic nerve damage in glaucoma.

## Introduction

The recent introduction of optical coherence tomography angiography (OCT-A) has sparked interest in evaluating vascular alterations in the retina and optic nerve head (ONH) for diagnosis, staging, and monitoring in glaucoma. OCT-A is an extension of OCT which allows non-invasive visualization of the retinal vasculature by detecting motion contrast from perfused blood vessels without the use of exogenous dye. In principle, OCT-A compares sequential B-scans acquired at the same location to detect change. As stationary structures would appear static in sequential B-scans, changes detected by OCT-A are largely attributed to erythrocyte movement in the perfused vasculatures. A number of algorithms such as split-spectrum amplitude decorrelation angiography (SSADA), OCT-A ratio analysis, and optical microangiography (OMAG) have been devised to compute blood flow measurements from the sequential B-scans ^1, 2^. Some of these measurements reported in the literature include vessel density (commonly annotated as the percentage of detected vessel area over the imaged area), flow index (a dimension-less parameter between 0 and 1 representing the average decorrelation signal), and blood flux index (the mean flow intensity in the vessel area normalized between 0 and 1 by dividing the full dynamic range of blood flow signal intensity). It is worth noting that these indexes are surrogate measures and their validity for measurement of blood flow remains to be investigated.

## Diagnostic performance of OCT-A measurements for glaucoma detection

Jia and colleagues provided the first account of vascular abnormalities at the ONH measured by a swept-source OCT in glaucoma ^3^. They showed that the optic disc flow index was reduced by 25% in glaucomatous eyes (0.161 ± 0.008) compared with healthy eyes (0.121 ± 0.026). Using a cut-off value of 0.1515, they showed that the sensitivity and specificity for the detection of glaucoma were both 100% (the visual field mean deviation in the glaucoma group was −3.28 ± 4.12 dB). The flow index was highly associated with visual field pattern standard deviation (R ^2^ = 0.752). The same group then evaluated the peripapillary flow index and the peripapillary vessel density for discrimination between glaucomatous and healthy eyes by using a spectral-domain OCT and reported the area under the receiver operating characteristic curve (AUC) to be 0.892 and 0.938, respectively ^4^. It remains controversial whether OCT-A measurements have a higher diagnostic performance for glaucoma detection compared with conventional OCT measurements such as the retinal nerve fiber layer (RNFL) thickness, neuroretinal rim width, and macular ganglion cell and inner plexiform layer thickness. Chen and colleagues demonstrated that the peripapillary blood flux index measured between the internal limiting membrane (ILM) and RNFL using OMAG and circumpapillary RNFL thickness had comparable diagnostic performance for the detection of glaucoma suspect (AUC = 0.76 versus 0.70, respectively) and glaucoma (AUC = 0.93 versus 0.97, respectively) ^5^. In a recent study, Rao and colleagues compared the diagnostic performance for glaucoma detection between OCT-A vessel density measurements using SSADA and OCT measurements (circumpapillary RNFL thickness, neuroretinal rim area, and ganglion cell complex [GCC]) ^6^. All vessel density measurements, including the radial peripapillary capillary (measured between the ILM and RNFL), the ONH segment vessel (measured from 2,000 µm above the ILM to 150 µm below the ILM), and the macular superficial plexus (between the ILM to the inner plexiform layer), were found to have significantly smaller AUCs compared with OCT measurements. Discrepancies among the studies are likely attributed to the different definitions adopted and varying stages of glaucoma patients included in the analysis.

## OCT-A abnormality in glaucoma: primary damage or secondary change?

Lee and colleagues hypothesized that if vascular abnormality were a consequence of optic nerve damage, it would be observed only at the area of RNFL defect ^7^. Examining 98 primary open-angle glaucoma eyes with a localized RNFL defect, the authors demonstrated that the radial peripapillary capillary vascular abnormality detected by OCT-A using SSADA exactly coincided with the RNFL defect in both the location and the extent, suggesting that vascular change is a consequence of optic nerve damage in glaucoma. On the other hand, Chen and colleagues studied the microvasculature density (excluding the effect of large retinal vessels) and blood flux index between the ILM and RNFL measured using OMAG at the peripapillary region in glaucomatous eyes with single-hemifield visual field defects and reported that the intact visual hemifield showed reduced blood flux index and microvasculature density in eyes with glaucoma compared with healthy eyes but that no significant difference in circumpapillary RNFL thickness between the groups was detected ^8^. While significant correlations between blood flow index/circumpapillary RNFL thickness and visual field mean deviation were observed in the normal visual hemifield in eyes with glaucoma, there was no correlation between microvasculature density/blood flux index and visual field mean deviation/circumpapillary RNFL thickness in the abnormal visual hemifield. Yarmohammadi and colleagues showed that while radial peripapillary capillary vessel density, macular superficial vessel density, RNFL thickness, and GCC thickness were all reduced in both the affected and the intact visual hemifields in eyes with glaucoma, the strength of association with visual field sensitivity measures was stronger for vessel density measurements using SSADA compared with RNFL and GCC thicknesses ^9^. Longitudinal studies investigating the temporal sequence of OCT-A and ONH/RNFL changes are needed to address whether the vascular changes detected by OCT-A are a cause or a consequence of optic nerve damage in glaucoma.

## Limitations of OCT-A

Motion artefacts and projection artefacts are common in OCT-A. A considerable proportion of OCT-A images remain suboptimal in quality for interpretation. For example, in a study evaluating the intra-visit and inter-visit variability of vessel density measurement in primary open-angle glaucoma and ocular hypertension patients, 78.3% of the participants were excluded because of suboptimal SSADA-derived OCT-A image quality ^10^. Poor-quality OCT-A scans are more common than poor-quality OCT scans. In a study in which both OCT-A and OCT measurements were performed by the same commercially available instrument, 17% and 29% of the OCT-A scans using SSADA were considered to have poor quality at the optic disc and the macula regions, respectively ^11^. By contrast, only 9% of OCT scans at the optic disc region and 3% of OCT scans at the macula were graded as poor quality. With the currently available OCT-A instruments, the scan time typically varies from 3 to 6 seconds for a 3×3 to 6×6 mm ^2^ scan ^12^, which is longer than imaging the ONH or the macula for RNFL and ganglion cell layer/inner plexiform layer analyses. The longer scan time in OCT-A can contribute to a higher incidence of motion artefact.

## Summary

Vascular abnormalities detected by OCT-A have been consistently observed in glaucoma. However, it remains unclear whether OCT-A provides additional diagnostic information for the detection of glaucoma compared with conventional OCT measurements such as circumpapillary RNFL thickness, neuroretinal rim width, and ganglion cell inner plexiform form layer thickness. Findings from the literature comparing OCT-A and OCT measurements for the detection of glaucoma and evaluation of structure function association are divergent. The temporal sequence of vascular changes and optic nerve damage in glaucoma remains to be elucidated.
